# Supplementary material for: A non-genetic switch triggers alternative telomere lengthening and cellular immortalization in ATRX deficient cells
Source: Nat Commun. 2023 Feb 20;14:939. doi: 10.1038/s41467-023-36294-6 (PMC9941109; doi:10.1038/s41467-023-36294-6)
Supplement: Supplementary file 1 — Supplementary Information [file 41467_2023_36294_MOESM1_ESM.pdf]

Supplementary Figure 1

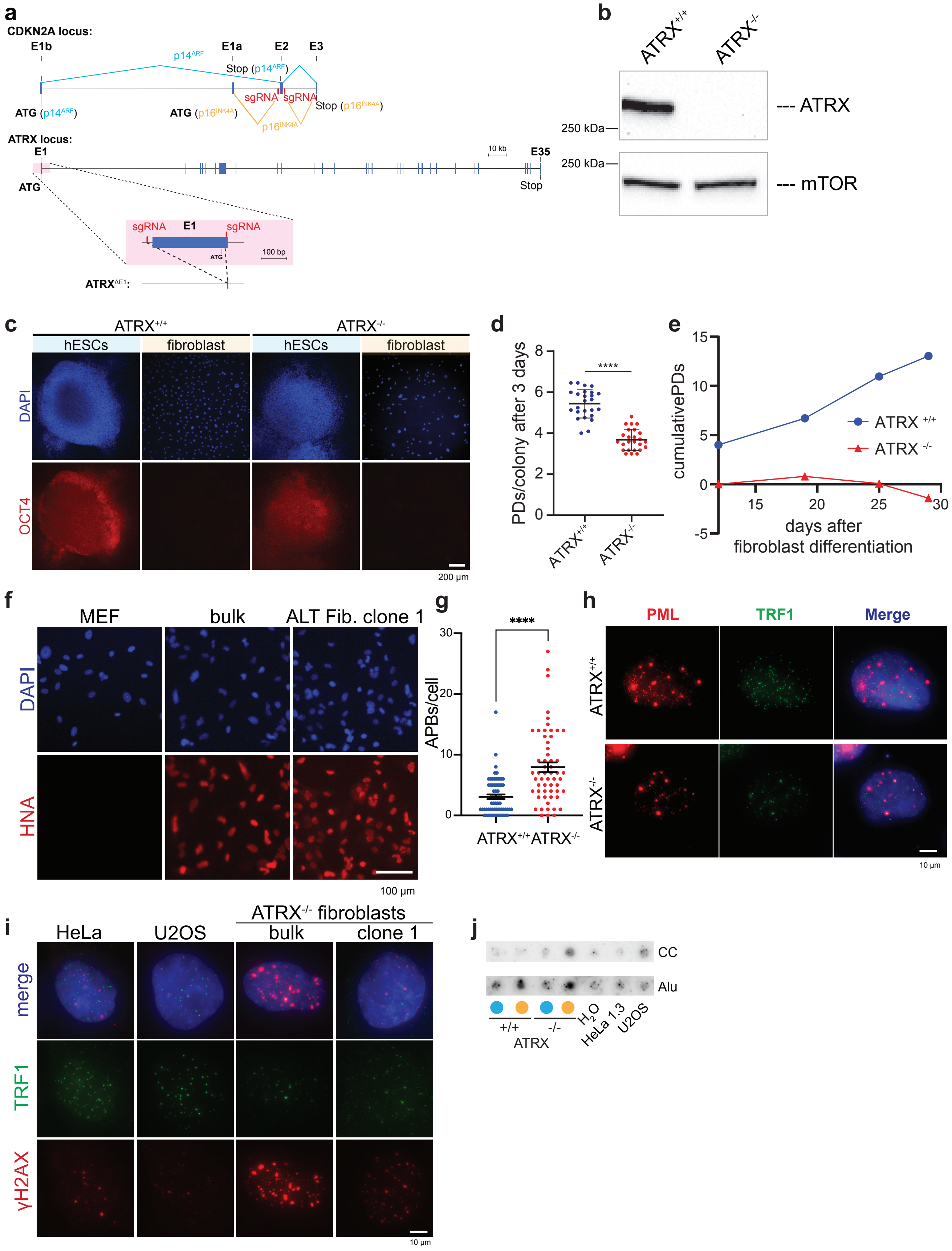

Supplementary Figure 1. Additional characterization of ATRX$^{-/-}$ cells.

A) Schematic of endogenous ATRX knockout (ATRX$^{-/-}$) in a CDKN2A knockout genetic background (CDKNA$^{-/-}$) (see Methods). Blue bars represent exons to scale, sgRNAs are indicated in red. Deletion of shared exon 2 (E2) in the CDKN2A locus leads to loss of both p14 and p16. Excision of exon 1 (E1) sequence between sgRNAs in the *ATRX* locus removes transcription and translation start sites.

(b) Western blot analysis of ATRX$^{+/+}$ and ATRX$^{-/-}$ cells. ATRX$^{-/-}$ cells do not express ATRX protein, mTOR serves as loading control. Source data are provided as a Source Data file.

(c) Differentiation of hESCs into fibroblasts resulted in loss of OCT4 pluripotency marker (red). Cells were counterstained with DAPI (blue). Both ATRX$^{+/+}$ and ATRX$^{-/-}$ lose OCT4 expression after differentiation into fibroblasts. Scale bar is 200 μm.

(d) Population doubling rate per colony (PDs/colony) of ATRX$^{+/+}$ (blue) and ATRX$^{-/-}$ (red), TERT$^{-/-}$ hESCs. Doublings were counted starting at 3 days after differentiation. ATRX $^{-/-}$ cells show a reduction of average PDs/colony, mean for ATRX$^{+/+}$ = 5.45, ATRX$^{-/-}$ = 3.68. Data shown are individual values (n = 25) with means ± s.d., asterisks represent p-value ($p < 0.0001$) as calculated by two-tailed Welch's t-test. Source data are provided as a Source Data file. Source data are provided as a Source Data file.

(e) Growth curve of ATRX$^{+/+}$ and ATRX$^{-/-}$ cells after differentiation and SV40 LT infection. Source data are provided as a Source Data file. Source data are provided as a Source Data file.

(f) Immunocytochemistry of ALT fibroblasts compared to mouse embryonic fibroblasts (MEFs). Proliferating ALT positive cells express human nuclear antigen (HNA, red). Cells were counterstained with DAPI. Scale bar is 100 μm.

(g) Quantification of colocalization of TRF1 and PML (APBs) in ATRX$^{+/+}$ and ATRX$^{-/-}$ cells fibroblasts after SV40 LT infection. ATRX$^{-/-}$ cells show a significant increase of the number of APBs per cell. ATRX$^{+/+}$: n = 63; ATRX$^{-/-}$: n = 57 over 1 experiment. Data shown are individual values with means ± s.d., asterisks represent p-value ($p < 0.0001$) as calculated by Kruskal-Wallis test. Source data are provided as a Source Data file. Source data are provided as a Source Data file.

(h) Maximum projection images for TRF1 (green) and PML (red) with DAPI (blue) counterstain. ATRX$^{-/-}$ fibroblasts show the presence of APBs when compared to their ATRX$^{+/+}$ counterpart. Scale bar is 10 μm.

(i) Maximum projection images stained for gH2AX (red) and TRF1 (green) with DAPI (blue) counterstain. ATRX$^{-/-}$ fibroblasts, either bulk cultures or single clone, show telomere dysfunction induced foci (TIFs).

(j) Representative image of C-circle assay in both hESCs (cyan dots) and E7 differentiated cells (yellow dots) for both ATRX$^{+/+}$ and ATRX$^{-/-}$ genotypes, HeLa and U2OS cells are used as negative and positive control respectively. The Alu probe is used as DNA loading control.

Supplementary Figure 2

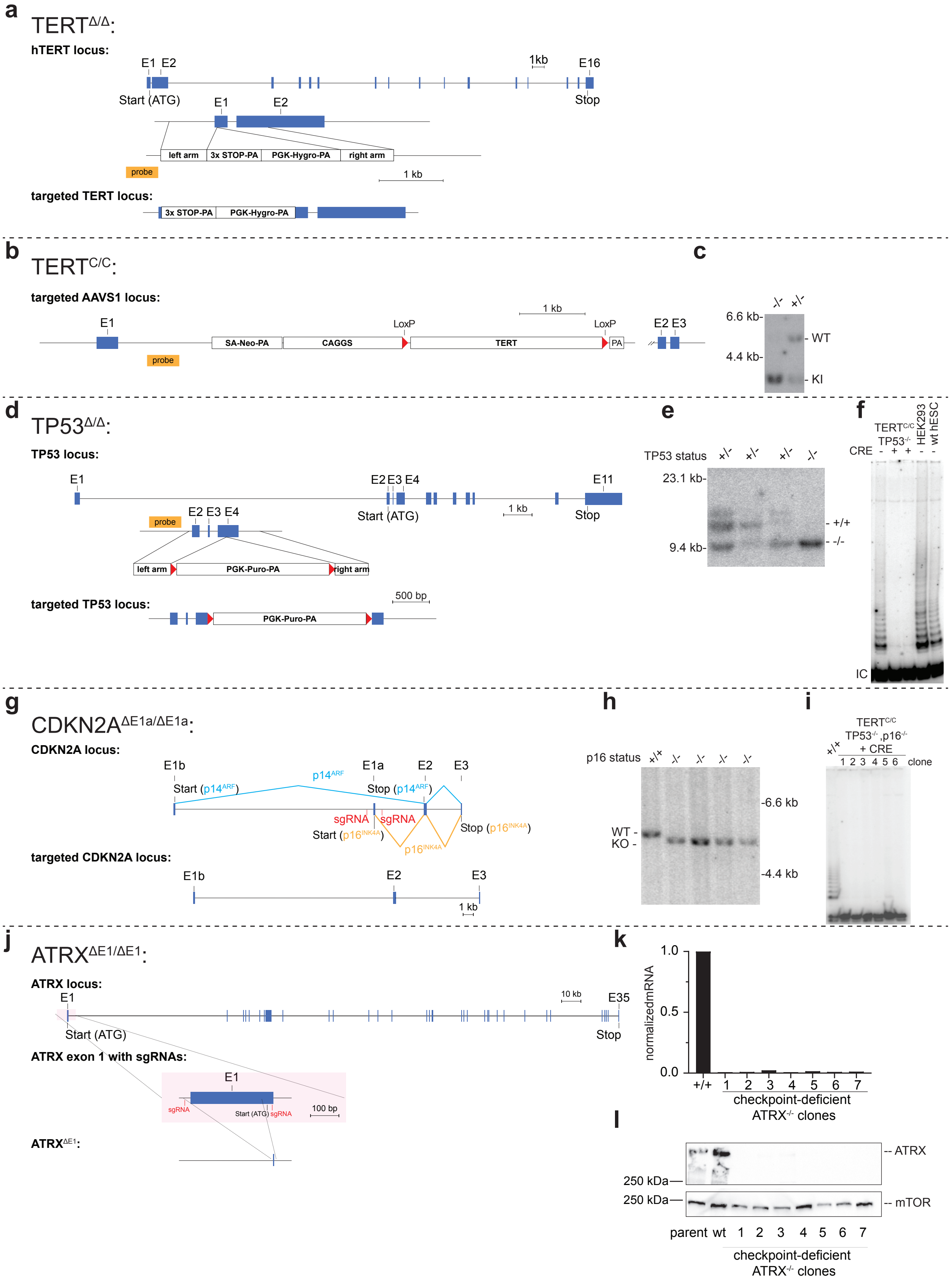

Supplementary Figure 2. Overview and genotyping of genetic engineering.

(a) Schematic of endogenous TERT knockout by biallelic insertion of a hygromycin resistance cassette.

(b) Schematic of biallelic loxP-flanked TERT insertion into AAVS1 loss.

(c) Southern blot confirmation of insertion.

(d) Schematic of endogenous TP53 knockout by biallelic insertion of a loxP-flanked puromycin resistance cassette.

(e) Southern blot confirmation of insertion.

(f) Cre recombinase-mediated removal of TERT from AAVS1 eliminates telomerase activity.

(g) Schematic of endogenous CDKN2A knockout by targeted biallelic excision of exon 1a encoding the translational start of p16.

(h) Southern blot confirmation of exon removal.

(i) *In vitro* telomerase repeat addition activity assay confirming ablation of active telomerase formation upon Cre-expression.

(j) Schematic of endogenous ATRX knockout ($ATRX^{-/-}$); excision of intermittent sequence between sgRNAs removes transcription and translation start sites.

(k) RT-qPCR confirmation of loss of ATRX mRNA expression in $ATRX^{-/-}$ clones.

(l) Western blot shows loss of ATRX protein expression in $ATRX^{-/-}$ clones. Source data are provided as a Source Data file.

Supplementary Figure 3

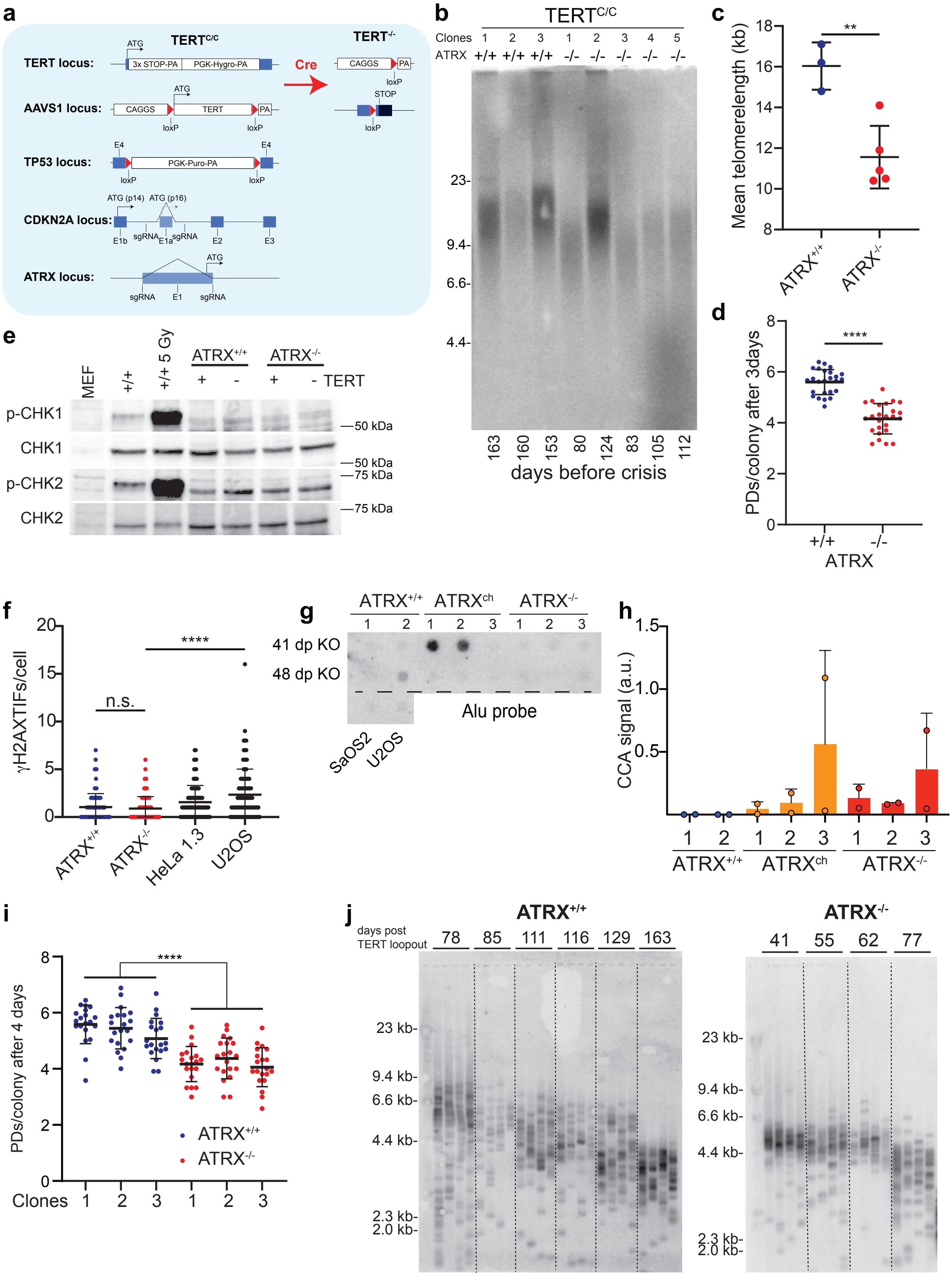

Supplementary Figure 3. Additional phenotypes of ATRX$^{-/-}$ TERT conditional ESCs.

(a) Overview of the genetic backgrounds of hESC lines obtained by targeted genome editing (see Methods). The tight control of *TERT* expression is achieved by knockout of the endogenous locus and targeted conditional overexpression from the AAVS1 locus. The *TP53* locus was knocked out by insertion of a PGK-Puromycin-polyA (PGK-Puro-PA) resistance cassette in the exon 4 flanked by loxP sites. Expression of Cre recombinase by mRNA delivery results in the elimination of *TERT* over-expression from the AAVS1 locus and loss of the PGK-Puro-PA cassette from the *TP53* locus. The locus remains ablated due to frameshift of inserted loxP site. *CDKN2A* locus is targeted to ablate exon E1a, causing selective p16 loss. ATRX$^{-/-}$ is obtained by targeting exon 1 with two flanking gRNAs causing collapse of the endogenous locus and loss of the ATG.

(b) Telomere restriction fragment assay of TERT$^{+/-}$ and TERT$^{C/C}$ hESCs. Plotted below are days in culture that TERT$^{C/C}$ hESCs proliferate following addition of Cre before telomere crisis eliminates all hESCs. DNA fragment sizes are indicated along the gel in kilobases.

(c) Quantification of mean telomere lengths (see methods) of ATRX$^{+/+}$ and ATRX$^{-/-}$ hESC clones shown in (b). Data shown are individual values for each clone with means ± s.d., asterisks represent p-value (= 0.0051) as calculated by two-tailed t-test. ATRX$^{+/+}$: n = 3; ATRX$^{+/+}$: n = 5. Source data are provided as a Source Data file. Source data are provided as a Source Data file.

(d) Population doubling rate of ATRX $^{+/+}$ and ATRX $^{-/-}$ TERT$^{C/C}$ hESCs. Data shown are individual values (n = 25) with means ± s.d., asterisks represent p-value (< 0.0001) as calculated by two-tailed t-test. Source data are provided as a Source Data file. Source data are provided as a Source Data file.

(e) Western blot analysis of DNA damage checkpoint activation. p-Chk1 and p-Chk2 are used to monitor DNA damage in ATRX $^{+/+}$ and ATRX $^{-/-}$ cells in TERT$^{c/c}$ or TERT$^{-/-}$. As a positive control, ATRX $^{+/+}$ cells were irradiated with 5 Gy. Source data are provided as a Source Data file. Source data are provided as a Source Data file.

(f) Quantification of γH2AX/telomere colocalizations (TIFs) per TERT$^{C/C}$ ES cell. ATRX$^{+/+}$: n = 117; ATRX$^{-/-}$: n = 117; HeLa: n = 92; U2OS: n = 94 over 1 experiment. Data shown are individual values with means ± s.d., . Asterisks represent p-value (< 0.0001) as calculated by Kruskal-Wallis test. Source data are provided as a Source Data file.

(g) Alu control membrane for C-circle assay of different ATRX$^{+/+}$, ATRX^{ch} (compound heterozygous) or ATRX$^{-/-}$ clones together with ATRX$^{+/+}$ cell lines, SaOS2 and U2OS.

(h) Quantification of C-circle assay. The signal is normalized on Alu and SaOS2. Data shown is means of two independent experiments (dots) for each clone. Bars represent means ± s.d., source data are provided as a Source Data file.

(i) Population doubling rate of different clones of ATRX$^{+/+}$ and ATRX$^{-/-}$ TERT$^{C/C}$ hESCs. Nuclei per colony for 20 colonies were counted for each genotype. Asterisks represent p-value (< 0.0001) as calculated by ordinary one-way ANOVA test. Source data are provided as a Source Data file.

(j) Single telomere length analysis (STELA) of XpYp telomere for ATRX$^{+/+}$ and ATRX$^{-/-}$ hESC lines following Cre addition and TERT loopout.

Supplementary Figure 4

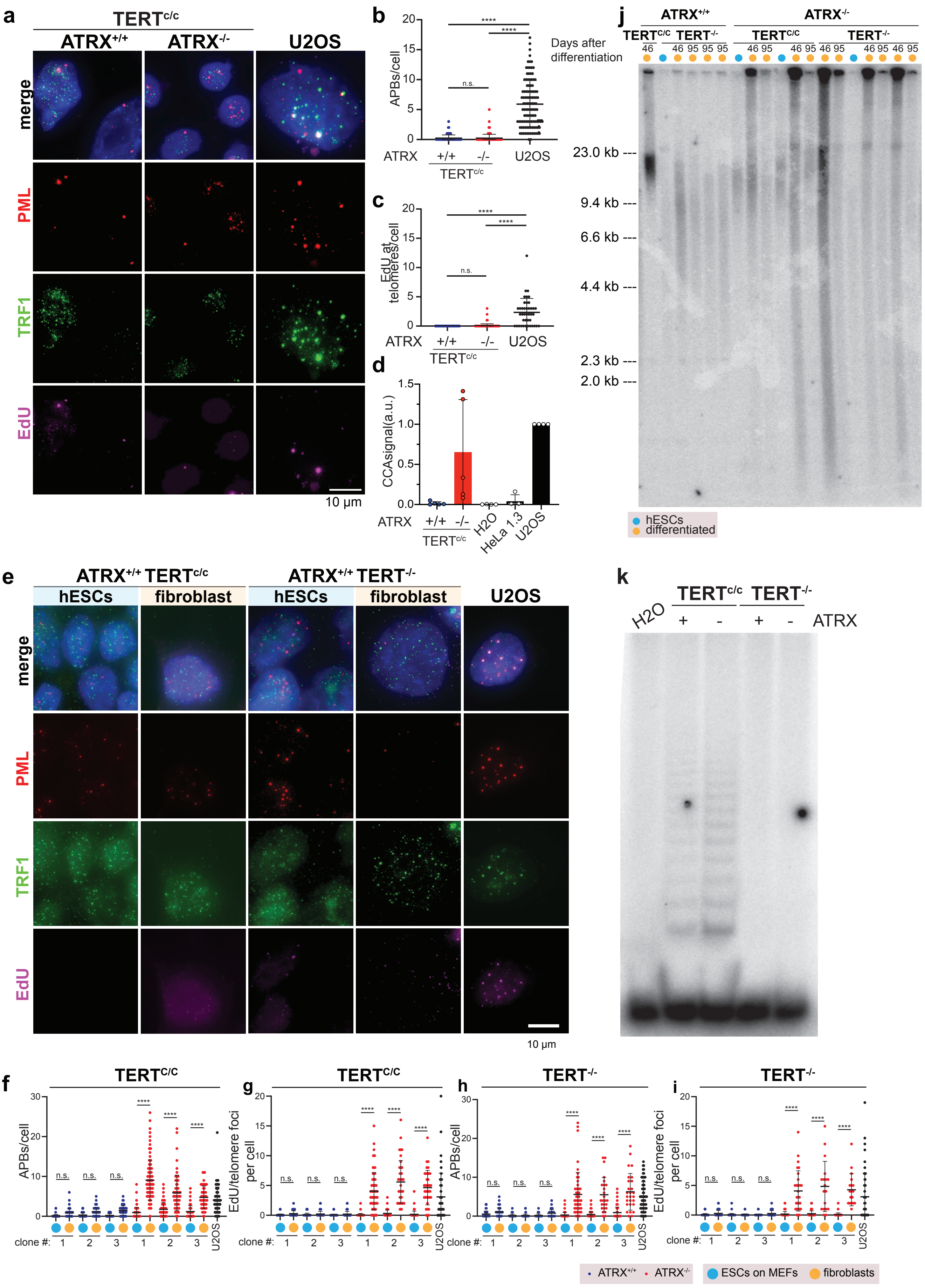

Supplementary Figure 4. Additional phenotypes of differentiated ATRX$^{-/-}$ cells.

(a) Maximum projection images stained for PML (red), TRF1 (green), and EdU (violet), with DAPI (blue) counterstain in ATRX$^{+/+}$, ATRX$^{-/-}$ TERT$^{c/c}$ cells. U2OS cells are used as a positive control fro the presence of APBs. Scale bar is 10 µm.

(b) Quantification of PML/telomere colocalizations (APBs) per ES cell. ATRX$^{+/+}$ (blue) and ATRX$^{-/-}$ (red) do not show a significant increase of APBs, U2OS cells (black) are used as positive control. ATRX$^{+/+}$: n = 184; ATRX$^{-/-}$: n = 187; U2OS: n = 111 over 1 experiment. Data shown are individual values with means ± s.d., asterisks represent p-value ($p < 0.0001$) as calculated by Kruskal-Wallis test. Source data are provided as a Source Data file.

(c) Quantification of EdU/telomere colocalizations per ES cell. ATRX$^{+/+}$ (blue) and ATRX$^{-/-}$ (red) do not a significant increase of APBs, U2OS cells (black) are used as control. ATRX$^{+/+}$: n = 164; ATRX$^{-/-}$: n = 113; U2OS: n = 41 over 1 experiment. Data shown are individual values with means ± s.d., asterisks represent p-value ($p < 0.0001$) as calculated by Kruskal-Wallis test. Source data are provided as a Source Data file.

(d) C-circle assay in hESCs. ATRX$^{-/-}$ cells (red bar) show an increase of C-circle signal when compared to ATRX$^{+/+}$ (blue bar) and HeLa 1.3 (black bar) as negative control. U2OS cells (black bar) have been used as positive control. Data represent means of independent subclones ± s.d., represented as dots. Source data are provided as a Source Data file.

(e) Maximum projection images of TERT$^{C/C}$ hESCs and fibroblasts stained for PML (red), TRF1 (green), and EdU (violet), with DAPI (blue) counterstain. Scale bar is 10 µm.

(F-I) Quantification of PML/telomere and EdU/telomere colocalizations (APBs) per TERT$^{C/C}$ and per TERT$^{-/-}$ cell. ATRX$^{+/+}$ (blue) cells do not show a significant increase of APBs in either cell state. Differentiated ATRX$^{-/-}$ (red) cells show a significant higher quantity of APBs compared to matched hESCs status. U2OS (black) are used as positive control. Data shown are individual values with means ± s.d., asterisks represent p-value ($p < 0.0001$) as calculated by ordinary one-way ANOVA between matched genotypes in the hESCs state vs differentiated state. (f) TERT$^{c/c}$, ATRX$^{+/+}$ clone 1: n = 121 for hESCs, n = 78 for differentiated cells; TERT$^{c/c}$, ATRX$^{+/+}$ clone 2: n = 105 for hESCs, n = 87 for differentiated cells; TERT$^{c/c}$, ATRX$^{+/+}$ clone 3: n = 91 for hESCs, n = 66 for differentiated cells; TERT$^{c/c}$, ATRX$^{-/-}$ clone 1: n = 110 for hESCs, n = 97 for differentiated cells; TERT$^{c/c}$, ATRX$^{-/-}$ clone 2: n = 105 for hESCs, n = 64 for differentiated cells; ATRX$^{-/-}$ clone 3: n = 105 for hESCs, n = 50 for differentiated cells; U2OS: n = 73. (g) TERT$^{c/c}$, ATRX$^{+/+}$ clone 1: n = 115 for hESCs, n = 61 for differentiated cells; TERT$^{c/c}$, ATRX$^{+/+}$ clone 2: n = 136 for hESCs, n = 75 for differentiated cells; TERT$^{c/c}$, ATRX$^{+/+}$ clone 3: n = 137 for hESCs, n = 63 for differentiated cells; TERT$^{c/c}$, ATRX$^{-/-}$ clone 1: n = 136 for hESCs, n = 75 for differentiated cells; TERT$^{c/c}$, ATRX$^{-/-}$ clone 2: n = 97 for hESCs, n = 44 for differentiated cells; ATRX$^{-/-}$ clone 3: n = 94 for hESCs, n = 36 for differentiated cells; U2OS: n = 52. (h) TERT$^{c/c}$, ATRX$^{+/+}$ clone 1: n = 142 for hESCs, n = 77 for differentiated cells; TERT$^{c/c}$, ATRX$^{+/+}$ clone 2: n = 120 for hESCs, n = 75 for differentiated cells; TERT$^{c/c}$, ATRX$^{+/+}$ clone 3: n = 116 for hESCs, n = 57 for differentiated cells; TERT$^{c/c}$, ATRX$^{-/-}$ clone 1: n = 130 for hESCs, n = 49 for differentiated cells; TERT$^{c/c}$, ATRX$^{-/-}$ clone 2: n = 104 for hESCs, n = 25 for differentiated cells; ATRX$^{-/-}$ clone 3: n = 103 for hESCs, n = 27 for differentiated cells; U2OS: n = 69. (i) TERT$^{c/c}$, ATRX$^{+/+}$ clone 1: n = 106 for hESCs, n = 66 for differentiated cells; TERT$^{c/c}$, ATRX$^{+/+}$ clone 2: n = 127 for hESCs, n = 70 for differentiated cells; TERT$^{c/c}$, ATRX$^{+/+}$ clone 3: n = 116 for hESCs, n = 53 for differentiated cells; TERT$^{c/c}$, ATRX$^{-/-}$ clone 1: n = 97 for hESCs, n = 29 for differentiated cells; TERT$^{c/c}$, ATRX$^{-/-}$ clone 2: n = 91 for hESCs, n = 18 for differentiated cells; ATRX$^{-/-}$ clone 3: n = 94 for hESCs, n = 18 for differentiated cells; U2OS: n = 57. Source data are provided as a Source Data file.

(j) Telomere restriction fragment assay of ATRX$^{+/+}$ and ATRX$^{-/-}$ cells before and after Cre-mediated TERT loopout. hESCs are represented as cyan dots, E7 differentiated cells are represented as yellow dots. Days after differentiation are reported above the gel. ATRX$^{+/+}$ cells show an expected shortening of telomeres along time after TERT loopout and differentiation. ATRX$^{-/-}$ cells show the appearance of heterogeneous telomeres after differentiation indiscriminately from the TERT status. DNA fragment sizes are indicated along the gel in kilobases units.

(k) *In vitro* telomerase repeat addition activity assay in differentiated TERT$^{c/c}$, TERT$^{-/-}$, ATRX$^{+/+}$ and ATRX$^{-/-}$ cells.

Supplementary Figure 5

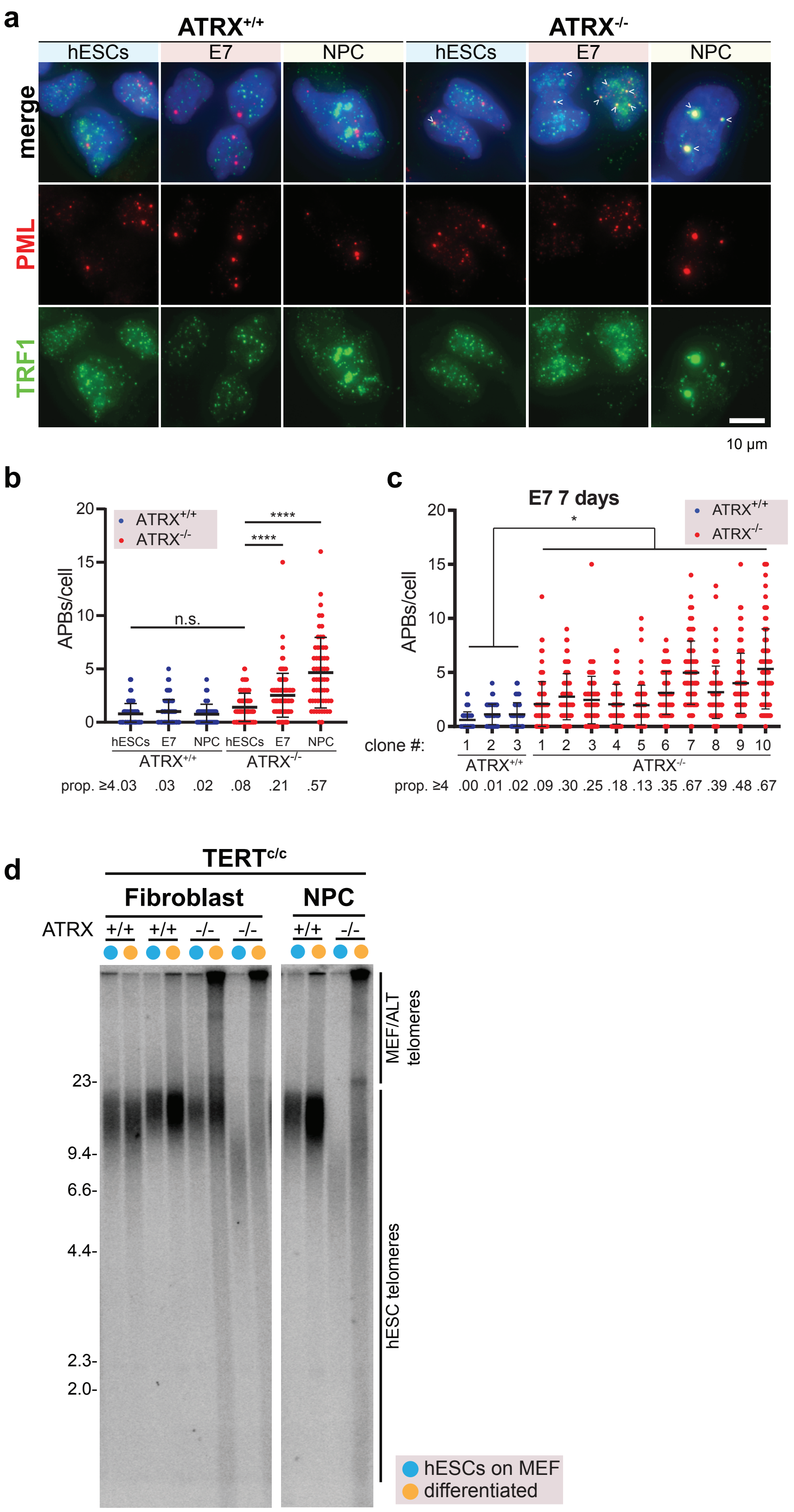

Supplementary Figure 5. Differentiation of ATRX$^{-/-}$ hESCs into multiple cell types also results in ALT.

(a) Maximum projection images of TERT$^{C/C}$ ATRX$^{+/+}$ and ATRX$^{-/-}$ cells stained for PML (red) and TRF1 (green) with DAPI (blue) counterstain after different differentiation protocols. Scale bar is 10 µm.

(b) Comparison of PML/telomere colocalizations (APBs) per cell in different cell types after differentiation for TERT$^{C/C}$ ATRX$^{+/+}$ and ATRX$^{-/-}$ cells. ATRX$^{+/+}$ cells show no significant increase of APBs/cells. ATRX$^{-/-}$ cells differentiated by different protocols show a significant increase of APBs/cell. ATRX$^{+/+}$: n = 122 for hESCs, n = 116 for E7, n = 46 for NPCs; ATRX$^{-/-}$: n = 104 for hESCs, n = 134 for E7, n = 58 for NPCs. Data shown are individual values with means ± s.d., asterisks represent p-value ($p < 0.0001$) as calculated by Kruskal-Wallis test. Source data are provided as a Source Data file.

(c) Quantification of PML/telomere colocalizations (APBs) per cell for TERT$^{C/C}$ ATRX$^{+/+}$ and ATRX$^{-/-}$ cells in multiple clones after E7 differentiation. Different clones of ATRX$^{+/+}$ cells show no significant increase of APBs, while 10 different clones of ATRX$^{-/-}$ cells show a significant increase of APBs. ATRX$^{+/+}$: n = 75 for clone 1, n = 85 for clone 2, n = 128 for clone 3; ATRX$^{-/-}$: n = 149 for clone 1, n = 118 for clone 2, n = 97 for clone 3, n = 95 for clone 4, n = 107 for clone 5, n = 97 for clone 6, n = 96 for clone 7, n = 122 for clone 8, n = 142 for clone 9, n = 79 for clone 10. Data shown are individual values with means ± s.d., asterisks represent p-value ($p < 0.01$) as calculated by two tailed t test on the mean values for each genotype. Source data are provided as a Source Data file.

(d) Telomere restriction fragment blot of different differentiation protocols of TERT$^{c/c}$, ATRX$^{+/+}$ and ATRX$^{-/-}$ cell lines. Two matched hESC (blue) and differentiated clones (orange) are represented side by side. ATRX$^{-/-}$ clones differentiated by different protocols show the appearance of heterogeneous telomeres when compared to the hESC and ATRX$^{+/+}$ counterpart. DNA fragment sizes are indicated along the gel in kilobases units.

Supplementary Figure 6

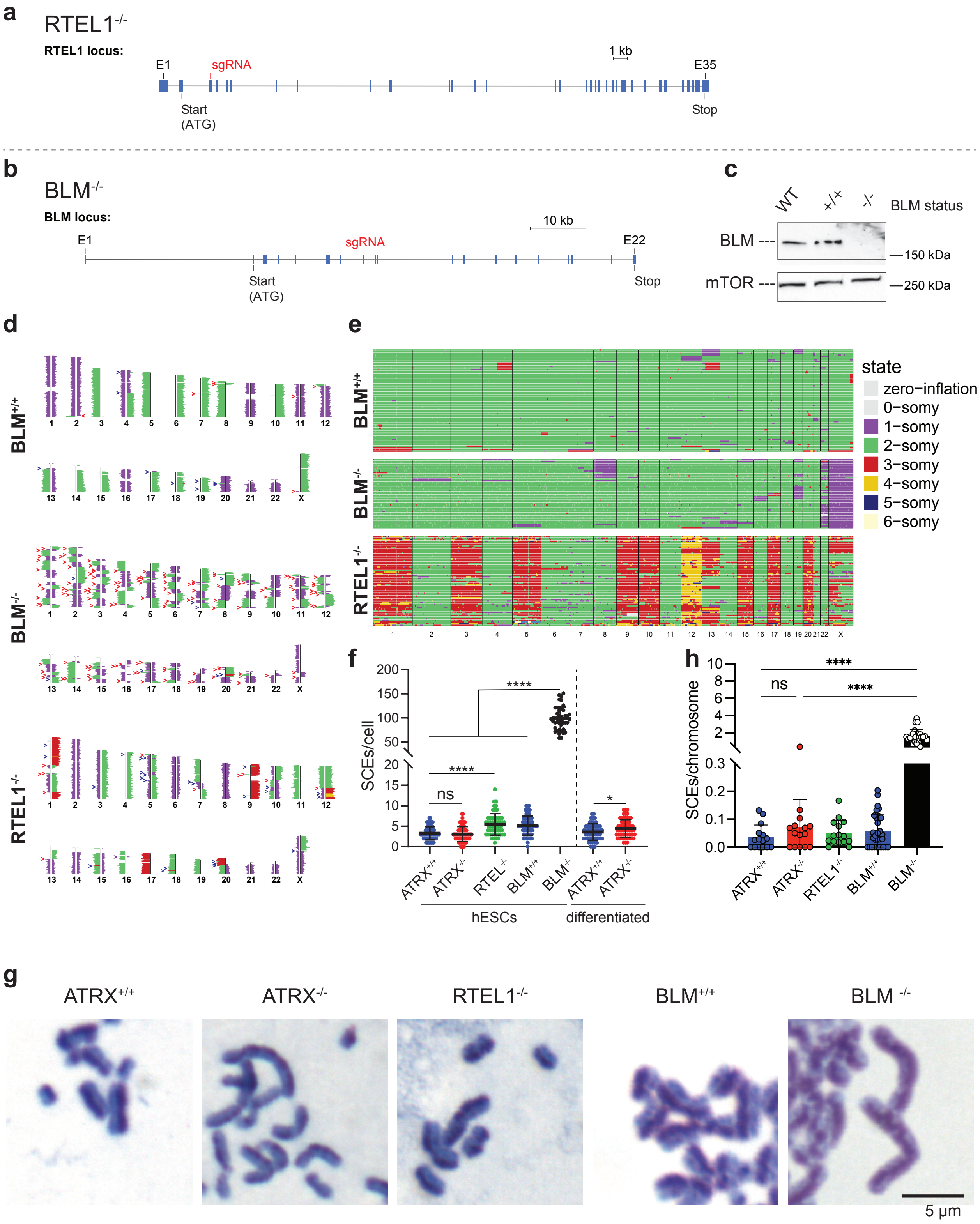

Supplementary Figure 6. Genomic instability by Strand-Seq

(a) and (b) Schematic of endogenous RTEL1 and BLM knockout (RTEL1 $^{-/-}$ and BLM $^{-/-}$ respectively). In both cases a single gRNA has been used to generate indels in the indicated coding exons.

(c) Western blot shows loss of BLM protein expression in BLM $^{-/-}$ hESCs. Source data are provided as a Source Data file.

(d) and (e) Strand-seq analysis of BLM $^{+/+}$, BLM $^{-/-}$ and RTEL1 $^{-/-}$ cells shows an increase in sister chromatid exchanges in BLM$^{-/-}$ and RTEL1 $^{-/-}$ cells as expected (van Wietmarschen *et al.*, 2018) and increased aneuploidy.

(f) Quantification of sister chromatid exchanges (SCEs) per cell show a significant increase of exchange events in ATRX$^{-/-}$ differentiated cells that was not detectable in the hESC state when compared to ATRX$^{+/+}$ cells. Dots represent individual values with means ± s.d, asterisks represent p value ($p < 0.0001$) as calculated by Kruskal-Wallis ANOVA test. ATRX$^{+/+}$ hESCs: n = 68; ATRX$^{-/-}$ hESCs: n = 68; RTEL1$^{-/-}$: n = 56; BLM$^{+/+}$: n = 63; BLM$^{-/-}$: n = 43; ATRX$^{+/+}$ differentiated: n = 53; ATRX$^{-/-}$ differentiated: n = 61. Source data are provided as a Source Data file.

(g) Harlequin chromosomes stain allows the detection of SCEs, BLM$^{-/-}$ shows an evident increase in sister chromatids exchanges.

(h) Quantification of sister chromatid exchanges (SCEs) per chromosome show a significant increase of exchange events in the positive control BLM$^{-/-}$. Exchanges per each chromosome were manually scored and quantified. Data shown are the sum of exchanges (bars), with means ± s.d.; each dot represents a sample, asterisks represent p values ($p < 0.0001$) as calculated by Kruskal-Wallis ANOVA test. ATRX$^{+/+}$: n = 15; ATRX$^{-/-}$: n = 15; RTEL1$^{-/-}$: n = 15; BLM$^{+/+}$: n = 30; BLM$^{-/-}$: n = 27. Source data are provided as a Source Data file.

Supplementary Figure 7

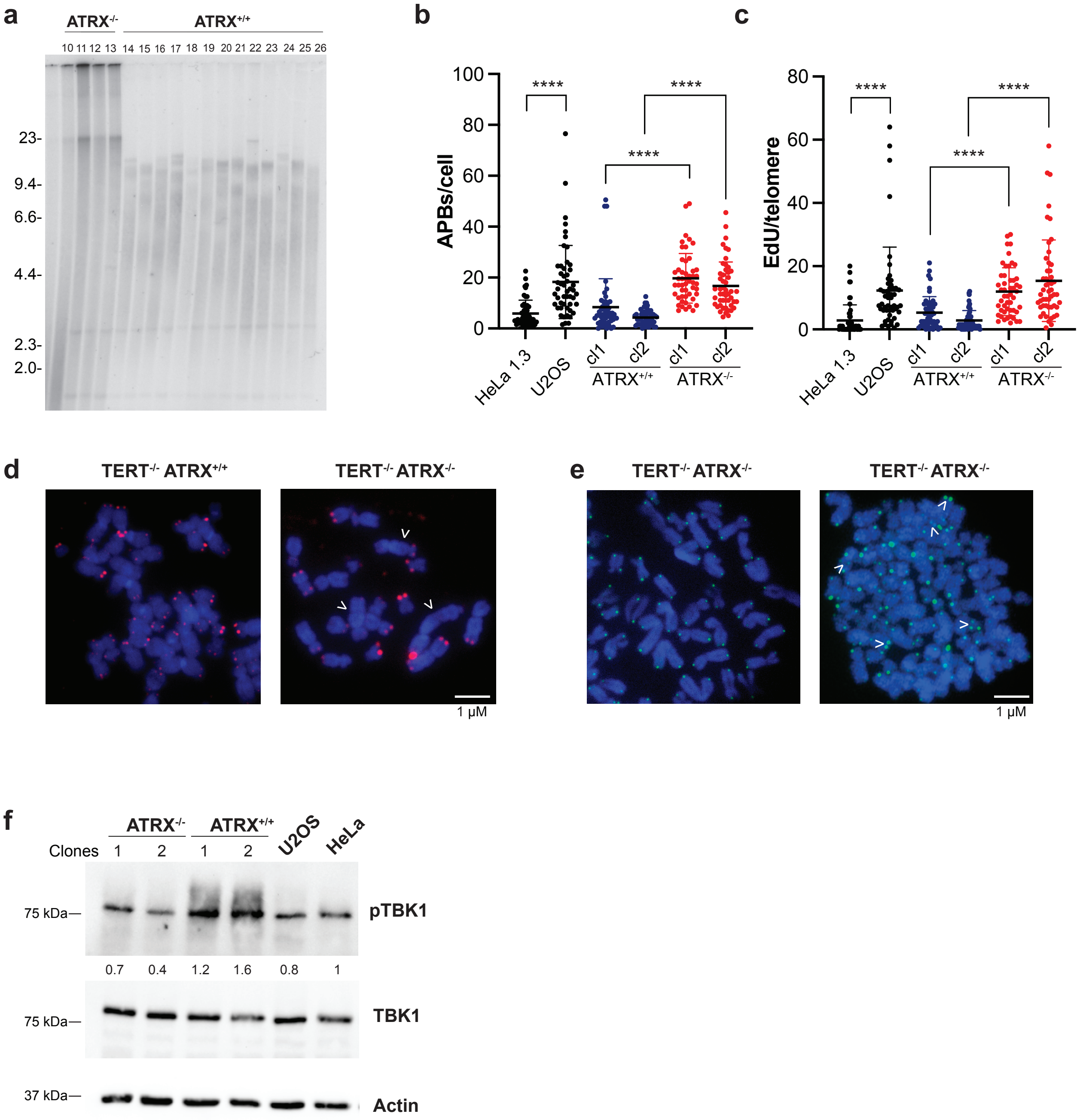

Supplementary Figure 7. Ongoing chromosomal instability is present in ALT positive cells

(a) Telomere restriction fragment blot of remaining ATRX$^{-/-}$ and ATRX$^{+/+}$ clones. DNA fragments sizes are indicated along the panel in kilobases.

(b) (c) Quantification of PML/TRF1 colocalization (APBs) and (c) EdU/telomere per cell. ≥ 40 nuclei are represented in this analysis. Data shown are individual values with means ± s.d., asterisks represent p values as calculated by two-way ANOVA ($p < 0.0001$); (b) HeLa: n = 107; U2OS n = 107; ATRX$^{+/+}$ clone 1: n = 92; ATRX$^{+/+}$ clone 2: n = 108; ATRX$^{-/-}$ clone 1: n = 107; ATRX$^{-/-}$ clone 2: n = 107; over 2 independent experiments. (c) HeLa: n = 107; U2OS n = 106; ATRX$^{+/+}$ clone 1: n = 92; ATRX$^{+/+}$ clone 2: n = 108; ATRX$^{-/-}$ clone 1: n = 107; ATRX$^{-/-}$ clone 2: n = 107; over 2 independent experiments. Source data are provided as a Source Data file.

(d) Maximum projection images of metaphase spreads in TERT$^{-/-}$, ATRX$^{+/+}$ and ATRX$^{-/-}$ fibroblasts. Metaphase spreads were obtained and stained for telomeres (red) (see Methods). Arrows point of identified chromosomal fusions.

(e) Single channel maximum projection image of CO-FISH stain in TERT-/-, ATRX-/- cells. Arrows indicated telomeric sister chromatid exchanges (T-SCE).

(f) Western blot analysis of cGAS-STING pathway activation. Protein extracts were probed for TBK1 phosphorylation (pTBK1) as a readout of cGAS-STING activation. Extracts were normalized for the loading control (Actin) and pTBK1 signal was corrected for the total TBK1 content. Source data are provided as a Source Data file.

Supplementary Figure 8

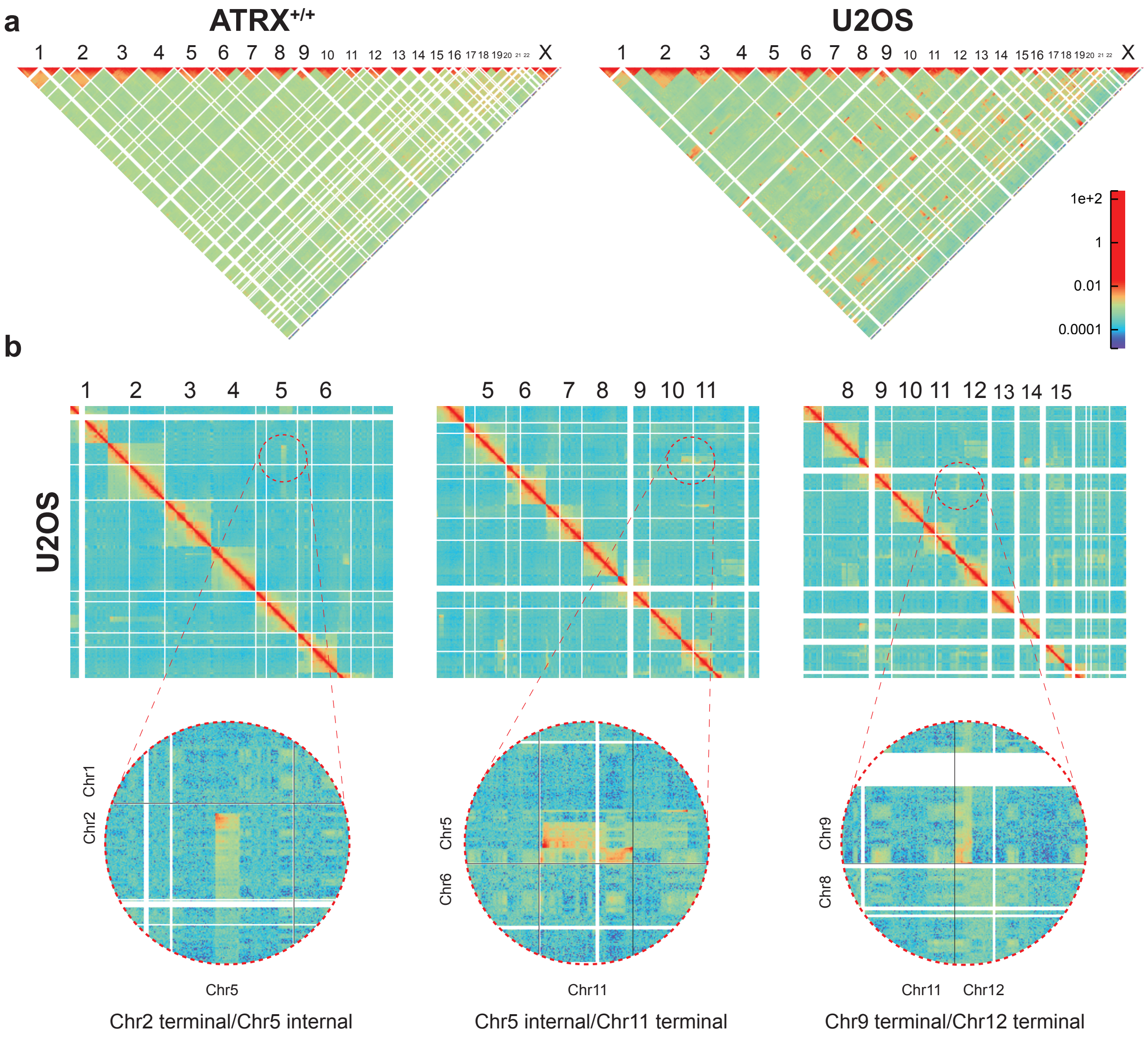

Supplementary Figure 8. Micro-C analysis shows continuous telomeric instability in ALT positive cells

(a) Micro-C whole genome chromosome maps of ATRX$^{+/+}$ clones and U2OS cells. Color bar represents the number of reads.

(b) Magnification of different Micro-C chromosome maps in U2OS cells. Dotted circles highlight contacts involving terminal chromosomal regions and magnifications of different contacts are also shown.

Supplementary Table 1: Cell lines used in this study

Line	Editing strategy	Editing outcome		
ATRX $^{+/+}$	*CDKN2A* exon 2 deletion	*ATRX* wild type		
ATRX $^{-/-}$		*ATRX* exon 1 deletion		
ATRX $^{+/+}$, TERT $^{c/c}$	conditional *TERT* overexpression from AAVS1 locus	*TP53* exon 4 disruption	*p16* exon E1a deletion	*ATRX* wild type
ATRX $^{-/-}$, TERT $^{c/c}$				*ATRX* exon 1 deletion
ATRX $^{+/+}$, TERT $^{-/-}$	*TERT* overexpression loss from AAVS1 locus by Cre recombination			*ATRX* wild type
RTEL1 $^{-/-}$, TERT $^{c/c}$	conditional *TERT* overexpression from AAVS1 locus	*TP53* exon 4 disruption	*p16* exon E1a deletion	*RTEL1* exon 3 disruption
BLM $^{-/-}$, TERT $^{c/c}$				*BLM* exon 8 disruption

Supplementary Table 2: Summary of gene knockouts

ATRX

Line	Strategy	Clones genotyped	Wildtype		(Compound) heterozygous		Knockout	
			#	%	#	%	#	%
TERT$^{C/C}$ cp-	exon 1 excision	264	204	77.3%	48	18.2%	12	4.5%
TERT$^{C/C}$ cp+	exon 1 excision	48	48	100%	0	0%	0	0%
TERT$^{+/-}$ cp-	exon 1 excision	72	61	84.7%	9	12.5%	2	2.8%
wildtype	exon 1 excision	42	42	100%	0	0%	0	0%

RTEL1

Line	Strategy	Clones genotyped	Wildtype		Heterozygous		Knockout	
			#	%	#	%	#	%
TERT$^{C/C}$ cp-	exon 3 NHEJ	24	21	87.5%	2	8.3%	1	4.2%

BLM

Line	Strategy	Clones genotyped	Wildtype		Heterozygous		Knockout	
			#	%	#	%	#	%
TERT$^{C/C}$ cp-	exon 3 NHEJ	94	89	94.7%	4	4.3%	1	1.1%

Supplementary Table 3: ATRX knockout targeting attempts

Line	Strategy	Clones genotyped	Wildtype		(Compound) heterozygous		Knockout	
			#	%	#	%	#	%
TERT$^{C/C}$ cp- att. 1	exon 1 excision	144	113	78.5%	24	16.7%	7	4.9%
TERT$^{C/C}$ cp- att. 2	exon 1 excision	24	10	41.7%	14	58.3%	0	0%
TERT$^{C/C}$ cp- att. 3	exon 1 excision	96	81	84.4%	10	10.4%	5	5.2%
TERT$^{C/C}$ cp+	exon 1 excision	48	48	100%	0	0%	0	0%
TERT$^{+/-}$ CDKN2A$^{-/-}$	exon 1 excision	72	61	84.7%	9	12.5%	2	2.8%
wildtype att. 1	exon 1 excision	8	8	100%	0	0%	0	0%
wildtype att. 2	exon 1 excision	34	34	100%	0	0%	0	0%

Supplementary Table 4: Chromosome abnormalities measured by FISH

	Fusion (without telomeric signal)	Fusion (with telomeric signal)	Chromosomes
ATRX$^{+/+}$ clone 1	0.004	0.000	254
ATRX$^{+/+}$ clone 2	0.016	0.004	251
ATRX$^{-/-}$ clone 1	0.084	0.042	263
ATRX$^{-/-}$ clone 2	0.038	0.019	265

Supplementary Table 5: Scoring of Hi-C contacts

TERT$^{-/-}$ ATRX$^{-/-}$ clone	Terminal – Terminal (chromosomes)	Terminal – Internal (chromosomes)	Internal – Internal (chromosomes)
**1**	*2-10, 6-18, 7-18, 10-12*	*2-4*	-
**2**	-	*2-4, 10-2, 12-10*	-
**3**	-	-	-
**4**	-	-	-
**5**	*4-5, 6-10*	-	-
**6**	*2-18, 5-11*	-	-
**7**	*8-10*	*10-2, 12-10*	-
**8**	*12-16*	*X-12, X-7*	-
**9**	*7-10*	-	-
**10**	*20-21*	-	-
**11**	*6-10*		*8-15*
**12**	*3-11, 4-5, 10-14*	*3-10, 6-7*	-
**13**	-	*2-4, 10-2, 12-10*	-
**14**	*3-11, 4-5, 10-14*	*3-10, 6-7*	-

Supplementary Table 6: gRNAs used in this study

sgRNA	Guide sequence	PAM
ATRX_KO1	gctttggaggaggtagccaa	tgg
ATRX_KO2	acatgaccgctgagcccatg	agg
BLM_KO	tctctatgagaggaagctct	tgg
CDKN2A_KO1	accattctgttctctctggc	agg
CDKN2A_KO2	cgcggaaggtccctcaggtg	agg
RTEL1_KO	tggcgagaacacctccgaga	cgg

Supplementary Table 7: antibodies used in this study

Target	Manufacturer	Cat. Number	Species	ICC	Immunoblot
ATRX	Sigma	HPA001906	Rabbit		1:500
BLM	Abcam	ab2179	Rabbit		1:1000
CHK1	Santa Cruz	sc-8408	Mouse		1:1000
CHK1 pS345	Cell Signaling	2348	Rabbit		1:2000
CHK2	Cell Signaling	3440	Mouse		1:1000
pCHK2 pT68	Cell Signaling	2661	Rabbit		1:2000
HNA	Abcam	ab191181	Mouse	1:1000	
mTOR	Cell Signaling	2983	Rabbit		1:1000
OCT4	Abcam	ab19857	Rabbit	1:2000	
PML	Santa Cruz	sc-966	Mouse	1:100	
TRF1	generous gift of Jan Karlseder		Rabbit	1:1000	
γH2A.X	Millipore	05-636	Mouse	1:1000	
TBK1	Cell Signaling	51872	Mouse		1:1000
pTBK1	Cell Signaling	5483	Rabbit		1:1000
beta-Actin	Sigma Aldrich	A5316	Mouse		1:2000
