# Supplementary material for: A non-genetic switch triggers alternative telomere lengthening and cellular immortalization in ATRX deficient cells
Source: Nat Commun. 2023 Feb 20;14:939. doi: 10.1038/s41467-023-36294-6 (PMC9941109; doi:10.1038/s41467-023-36294-6)
Supplement: Supplementary file 3 — Reporting Summary [file 41467_2023_36294_MOESM3_ESM.pdf]

nature portfolio

Corresponding author(s): Dirk Hockemeyer

Last updated by author(s): Dec 19, 2022

Reporting Summary

Nature Portfolio wishes to improve the reproducibility of the work that we publish. This form provides structure for consistency and transparency in reporting. For further information on Nature Portfolio policies, see our Editorial Policies and the Editorial Policy Checklist.

Statistics

For all statistical analyses, confirm that the following items are present in the figure legend, table legend, main text, or Methods section.

n/a	Confirmed	
☐	☒	The exact sample size (*n*) for each experimental group/condition, given as a discrete number and unit of measurement
☐	☒	A statement on whether measurements were taken from distinct samples or whether the same sample was measured repeatedly
☐	☒	The statistical test(s) used AND whether they are one- or two-sided<br>*Only common tests should be described solely by name; describe more complex techniques in the Methods section.*
☒	☐	A description of all covariates tested
☒	☐	A description of any assumptions or corrections, such as tests of normality and adjustment for multiple comparisons
☐	☒	A full description of the statistical parameters including central tendency (e.g. means) or other basic estimates (e.g. regression coefficient) AND variation (e.g. standard deviation) or associated estimates of uncertainty (e.g. confidence intervals)
☐	☒	For null hypothesis testing, the test statistic (e.g. *F*, *t*, *r*) with confidence intervals, effect sizes, degrees of freedom and *P* value noted<br>*Give P values as exact values whenever suitable.*
☒	☐	For Bayesian analysis, information on the choice of priors and Markov chain Monte Carlo settings
☒	☐	For hierarchical and complex designs, identification of the appropriate level for tests and full reporting of outcomes
☒	☐	Estimates of effect sizes (e.g. Cohen's *d*, Pearson's *r*), indicating how they were calculated

Our web collection on statistics for biologists contains articles on many of the points above.

Software and code

Policy information about availability of computer code

Data collection	Software used for data collection: NIS-Elements 4.51.00 64-bit; Zeiss Zen 2012 (blue edition) 64-bit
Data analysis	Software used for data analysis: GraphPad Prism 8, Microsoft Excel, for Strand-seq and Micro-C tools used for the analysis are reported in the respective sections in "Methods" section.

For manuscripts utilizing custom algorithms or software that are central to the research but not yet described in published literature, software must be made available to editors and reviewers. We strongly encourage code deposition in a community repository (e.g. GitHub). See the Nature Portfolio guidelines for submitting code & software for further information.

Data

Policy information about availability of data

All manuscripts must include a data availability statement. This statement should provide the following information, where applicable:

- Accession codes, unique identifiers, or web links for publicly available datasets
- A description of any restrictions on data availability
- For clinical datasets or third party data, please ensure that the statement adheres to our policy

Hi-C data are available at the Gene Expression Omnibus under accession number GSE212809.

March 2021

Human research participants

Policy information about studies involving human research participants and Sex and Gender in Research.

Reporting on sex and gender	NA
Population characteristics	NA
Recruitment	NA
Ethics oversight	NA

Note that full information on the approval of the study protocol must also be provided in the manuscript.

Field-specific reporting

Please select the one below that is the best fit for your research. If you are not sure, read the appropriate sections before making your selection.

☒ Life sciences ☐ Behavioural & social sciences ☐ Ecological, evolutionary & environmental sciences

For a reference copy of the document with all sections, see nature.com/documents/nr-reporting-summary-flat.pdf

Life sciences study design

All studies must disclose on these points even when the disclosure is negative.

Sample size	Sample sizes were predetermined for each experiment but no statistical calculation was applied to determine such size. Previous works have guided us in the determination of a sufficient sample size for each experimental condition. eg<br>https://doi.org/10.1091/mbc.E19-08-0447 ;<br>DOI: 10.1126/science.aao0535<br>doi: 10.1038/nsmb.2754<br>https://doi.org/10.1371/journal.pbio.3000594
Data exclusions	No data were excluded from the analysis.
Replication	Each experiment is the result of multiple replicates as indicated in the text. Where replicates were not possible, data are collected from multiple isolated clones/conditions. Attempts at replication were successful and such replicates are reported in this work.
Randomization	No randomization was performed.
Blinding	Immunofluorescence and metaphase spreads images were blinded to group allocation during prior to data analysis. Micro-C experiments were performed by blinding group allocation to the investigator both during data collection and analysis.

Reporting for specific materials, systems and methods

We require information from authors about some types of materials, experimental systems and methods used in many studies. Here, indicate whether each material, system or method listed is relevant to your study. If you are not sure if a list item applies to your research, read the appropriate section before selecting a response.

Materials & experimental systems

n/a	Involved in the study	
☐	☒	Antibodies
☐	☒	Eukaryotic cell lines
☒	☐	Palaeontology and archaeology
☒	☐	Animals and other organisms
☒	☐	Clinical data
☒	☐	Dual use research of concern

Methods

n/a	Involved in the study	
☒	☐	ChIP-seq
☒	☐	Flow cytometry
☒	☐	MRI-based neuroimaging

Antibodies

Antibodies used	ATRX, Sigma, HPA001906, polyclonal

	BLM, Abcam, ab2179, polyclonal;<br>CHK1, Santa Cruz Biotechnology, sc-8408, G-4 monoclonal;<br>CHK1 pS345, Cell Signaling, 2348, monoclonal 133D3;<br>CHK2, Cell Signaling, 3440, monoclonal 1C12;<br>CHK2 pT68, Cell Signaling, 2661, polyclonal;<br>HNA, Abcam, ab191181, monoclonal 235-1;<br>mTOR, Cell Signaling, 2938, monoclonal 7C10;<br>OCT4, Abcam, ab19857, polyclonal;<br>PML, Santa Cruz Biotechnology, sc-966, monoclonal PG-M3;<br>TRF1, generous gift of Jan Karlseder, polyclonal, not commercially available;<br>γH2A.X, Millipore, 05-636, monoclonal JBW301;<br>TBK1, Cell Signaling, 51872, monoclonal E9H5S;<br>pTBK1, Cell Signaling, 5483, monoclonal D52C2<br>beta-Actin, Sigma Aldrich, A5316, monoclonal AC-74
Validation	ATRX, Sigma, HPA001906, polyclonal: orthogonal RNA-seq; siRNA; IHC, ICC, western blot, immunofluorescence<br>BLM, Abcam, ab2179, polyclonal: Western blot, Immunoprecipitation;<br>CHK1, Santa Cruz Biotechnology, sc-8408, G-4 monoclonal: Western blot, IHC, Immunofluorescence<br>CHK1 pS345, Cell Signaling, 2348, monoclonal 133D3: Western blot, Immunofluorescence, Flow cytometry<br>CHK2, Cell Signaling, 3440, monoclonal 1C12: Western blot, IHC, Immunofluorescence<br>CHK2 pT68, Cell Signaling, 2661, polyclonal: Western blot, Immunoprecipitation, Flow cytometry<br>HNA, Abcam, ab191181, monoclonal 235-1: Immunocytochemistry, Flow cytometry, Immunohistochemistry in frozen sections<br>mTOR, Cell Signaling, 2938, monoclonal 7C10: Western blot, siRNA, Immunoprecipitation, IHC, Immunofluorescence, Flow cytometry<br>OCT4, Abcam, ab19857, polyclonal: Immunofluorescence, Western blot, Immunoprecipitation<br>PML, Santa Cruz Biotechnology, sc-966, monoclonal PG-M3: Immunofluorescence, Western Blot, IHC,<br>TRF1, generous gift of Jan Karlseder, polyclonal, not commercially available:<br>γH2A.X, Millipore, 05-636, monoclonal JBW301: Immunofluorescence, Western blot<br>TBK1, Cell Signaling, 51872, monoclonal E9H5S: Western blot, knockout HCT116 cell line, Immunoprecipitation<br>pTBK1, Cell Signaling, 5483, monoclonal D52C2: Western blot, Immunofluorescence, Flow Cytometry<br>beta-Actin, Sigma Aldrich, A5316, monoclonal AC-74: Western blot, IHC, Immunofluorescence

Eukaryotic cell lines

Policy information about cell lines and Sex and Gender in Research

Cell line source(s)	WIBR3 hESCs, NIH stem cell registry #0079, RRID:CVCL_9767;<br>HeLa 1.3 cervical carcinoma cells were a gift of T. de Lange (The Rockefeller University, New York, NY);<br>U2OS and Saos-2 osteosarcoma cells were obtained from the UC Berkeley Cell Culture Facility.
Authentication	hESCs have been authenticated by Array CGH Fingerprint by WiCell and by whole genome sequencing. U2OS and SaOS-2 osteosarcoma cells were authenticated by the Berkeley Cell Culture facility using short tandem repeat DNA profiling (STR profiling). Further details on the cell lines used in this study can be found in the "Methods" section.
Mycoplasma contamination	Cells tested negative for Mycoplasma contamination by PCR assay. The test was run monthly on every cell line used in this study.
Commonly misidentified lines<br>(See ICLAC register)	No misidentified cell lines were used in the study.
